# Measurement of Vibrating Tympanic Membrane in an In Vivo Mouse Model Using Doppler Optical Coherence Tomography

**DOI:** 10.3390/jimaging5090074

**Published:** 2019-09-04

**Authors:** Deokmin Jeon, Joon Ki Kim, Mansik Jeon, Jeehyun Kim

**Affiliations:** 1 School of Electronics Engineering, College of IT Engineering, Kyungpook National University, 80 Daehak-ro, Buk-gu, Daegu 41566, Korea (D.J.) (J.K.); 2 Gumi R&D Center, LIG Nex1 Co., Ltd., 354-25, Sanho-daero, Gumi, Gyeongsangbuk-do 39262, Korea

**Keywords:** optical coherence tomography, Doppler measurement, middle ear

## Abstract

Optical coherence tomography (OCT) has a micro-resolution with a penetration depth of about 2 mm and field of view of about 10 mm. This makes OCT well suited for analyzing the anatomical and internal structural assessment of the middle ear. To study the vibratory motion of the tympanic membrane (TM) and its internal structure, we developed a phase-resolved Doppler OCT system using Kasai’s autocorrelation algorithm. Doppler optical coherence tomography is a powerful imaging tool which can offer the micro-vibratory measurement of the tympanic membrane and obtain the micrometer-resolved cross-sectional images of the sample in real-time. To observe the relative vibratory motion of individual sections (malleus, thick regions, and the thin regions of the tympanic membrane) of the tympanic membrane in respect to auditory signals, we designed an experimental study for measuring the difference in Doppler phase shift for frequencies varying from 1 to 8 kHz which were given as external stimuli to the middle ear of a small animal model. Malleus is the very first interconnecting region between the TM and cochlea. In our proposed study, we observed that the maximum change in Doppler phase shift was seen for the 4 kHz acoustic stimulus in the malleus, the thick regions, and in the thin regions of the tympanic membrane. In particular, the vibration signals were higher in the malleus in comparison to the tympanic membrane.

## 1. Introduction

In the field of otolaryngology, some of the most widely known diseases are effusion of otitis media, noise induced hearing loss, acute and chronic suppurative otitis media, Meniere’s disease, and perforated eardrum [1,2]. Most of these diseases are partially or completely related to the infection/damaged tympanic membrane (TM). Recently, laser Doppler vibrometry (LDV) has been adopted as the gold standard method for non-contact vibration measurement in various fields [3]. LDV has also been used as the non-contact gold standard method to diagnose TM vibration in otolaryngology [4]. The LDV method has a low signal-to-noise ratio, and also cannot provide high-resolution depth profile information due to the long coherence length of the laser. To overcome these shortcomings, we proposed a Doppler optical coherence tomography (DOCT) system. DOCT technology is an application of the OCT system that can be used by only adding phase calculation software without changing the OCT system hardware. Recently, optical coherence tomography (OCT) has been actively studied not only in ophthalmology [5,6,7,8] but also in medical fields such as dermatology [9,10,11] and otorhinolaryngology [12,13,14,15,16], and also has industrial [17,18,19,20,21] and agricultural [22,23] applications. Interest in the field of science that employs DOCT for the study of hearing mechanisms is increasing [24]. In addition, the resolution and sensitivity of DOCT has been sufficiently improved to provide middle ear structure and vibration information, and is being used in various studies on sound transmission [25,26,27,28]. 

Recently, parallel signal processing technology using a general-purpose graphics processing unit (GP-GPU) has been effective with equations requiring a considerable amount of computation. GP-GPU computing performance exceeds the central processing unit (CPU) computing speed of systems that process large amounts of data because it uses thousands of cores. In addition, in a real-time imaging system, image processing that is faster than scanning or image acquisition can have a stable frame rate by the clock signal of the hardware, so that the time interval between frames can be fixed. The continuous and stable time interval between frames is a very important parameter when correlation between images is required [29,30]. 

Through this study, we have proposed a DOCT system using Kasai’s autocorrelation method to measure the vibration tendency of TM in accordance with pure tone audio signals. Kasai’s autocorrelation algorithm uses the spatial averaging window to remove sensitive phase noise, which may reduce the speed of the system. Hence, by utilizing GPU-based spatial averaging, we have achieved a very effective means to maintain a stable frame rate [31,32].

## 2. Materials and Methods

**Figure 1:**
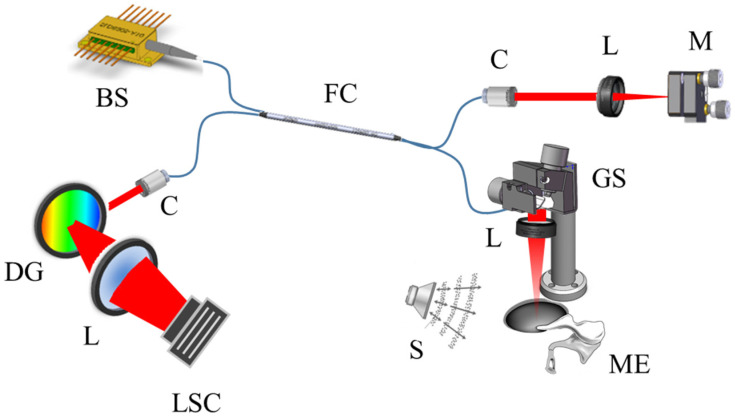
Schematic diagram of the DOCT (Doppler optical coherence tomography) system. BS: broadband source; C: collimator; DG: diffraction grating; FC: fiber coupler; GS: galvanometer scanner; L: lens; LSC: line scan camera; M: mirror; ME: middle ear; S: speaker.

Figure 1 shows the schematic diagram of the DOCT system. A superluminescent diode of wavelength centered at 850 nm and a 55 nm bandwidth (Exalos Ltd., Schlieren, Switzerland) was used as the light source of the interferometer. The light was transmitted through the sample and reference arms through a 1:1 fiber coupler. The sample arm consisted of a collimator, a 2-channel galvanometer scanner (GVS002, Thorlabs, Newton, NJ, USA), a focusing lens, and a loudspeaker for vibration stimulation. The reference arm consisted of a collimator, a focusing lens, and a mirror. The source power was 14 mW and the output power measured in the sample arm end (laser power exposed onto the sample surface) was 7 mW. The scattered light from the sample and the light reflected from the reference mirror combined to form an interference pattern. Each wavelength signal decomposed from the Fourier optical system was obtained from a line scan camera. The OCT images were acquired from the wavelength signals by performing a fast Fourier transform using a graphics processing unit (GeForce GTX 480, 700 MHz clock rate, 480 compute unified device architecture processors, NVIDIA; Santa Clara, CA, USA). 

All the animal experiments were performed in accordance with Kyungpook National University’s Institutional Animal and Human Care and Use Committee’s regulations on mice (No. KNU-2018-0100). A total of five healthy mice specimens were used in this study. All specimens used were ICR male mice which were five weeks old with masses of ~30 g. For each specimen, both the left and right ear’s tympanic membrane was imaged using the proposed system. In total, there were 10 samples. All mice samples were anesthetized using an isoflurane machine prior to imaging. During the experiment, oxygen mixed with 1% isoflurane was supplied to the respiratory organs through a commercially available medical breathing mask connected through a tube which was placed near the nose region of mice specimens. After respective injections of lidocaine HCl (2%) and epinephrine (1:80,000) around the external auditory canal, the skin and cartilage of the canal were removed to expose the whole TM. 

The DOCT image was obtained by calculating the phase difference of two A-lines. We measured the vibration using the DOCT while stimulating the TM with pure tone sounds. Outputs of 1, 2, 4, and 8 kHz were applied using a loudspeaker placed 30 cm away from the TM. The loudspeaker had a power output of 15 W and a measured sound volume of 92 dB. We had previously utilized a similar optical setup and DOCT algorithm for blood flow measurement in blood vessels for a small animal model which had been inoculated with squamous cell carcinoma [31], and for in vivo vibration measurement of middle ear structure [12].

**Figure 2:**
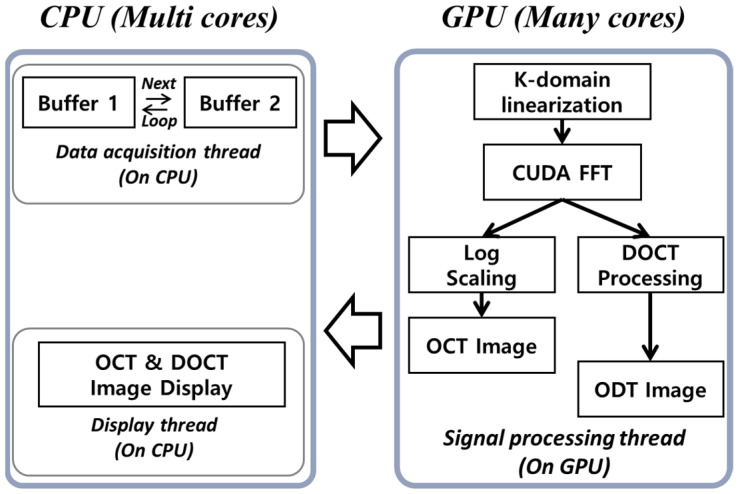
Operation algorithm of the DOCT system using both the multithreading on CPU (central processing unit) and parallel processing on the GPU (graphics processing unit).

Figure 2 shows the DOCT system software algorithm that controls the hardware modules using a multithreading algorithm for acquiring the raw signal, copy, and transfer of processed signals and image data output, respectively. In conventional data processing algorithms, the data acquisition thread and signal processing thread communicate with the main memory simultaneously, which leads to buffering and time delay for accessing the processed data for executing the Doppler phase estimation thread. To avoid this, we used a ring buffer structure in which the raw signals were sequentially written to two different allocated memories. The raw signal copied to the GPU memory was calculated by K-linearization, with background adjustment for noise filtering, fast Fourier transformation (FFT), log scaling, and Doppler frequency shift, by comparing phases from the two A-lines. The parallel signal processing using GPU was needed to obtain a constant speed Doppler signal. Doppler signal processing has a large amount of computation, and the signal processing speed cannot estimate the Doppler shift due to the time interval because each frame processing speed continuously changes. Since the line period of the line scan camera has a constant speed, it needs a faster signal processing speed than the camera frame rate in order to depend on the speed. Therefore, software has been developed using ultra-high-speed parallel signal processing technology using GPU.

The Doppler shift is calculated by the phase difference between the two A-lines. The phase information is derived by I (in-phase) and Q (quadrature phase) of the FFT operation. The Doppler shift $f_{D}$ induced by the vibrating TM is expressed as

(1) $$f_{D}=\frac{f_{a}}{2\pi }\tan^{-1}\left\{\frac{\frac{1}{M\left(N-1\right)}\sum_{m=1}^{M}\sum_{n=1}^{N-1}\left(Q_{m,n}I_{m,n+1}-I_{m,n}Q_{m,n+1}\right)}{\frac{1}{M\left(N-1\right)}\sum_{m=1}^{M}\sum_{n=1}^{N-1}\left(Q_{m,n}Q_{m,n+1}+I_{m,n}I_{m,n+1}\right)}\right\}.$$

In Equation (1), which is from [18], $f_{a}$ is the time interval of the camera acquisition between two A-lines, and the equation in the arctangent is the phase difference between two A-lines. In addition, M and N are the spatial averaging mask. The velocity *v* of the vibrating TM is expressed as

(2) $$v=\frac{\lambda_{0}f_{D}}{2n_{t}\cos\theta }.$$

Here,$\lambda_{0}$ is the center wavelength, $n_{t}$ is the refractive index, and θ is the angle between the TM and the direction of the sample arm beam.

## 3. Results

**Figure 3:**
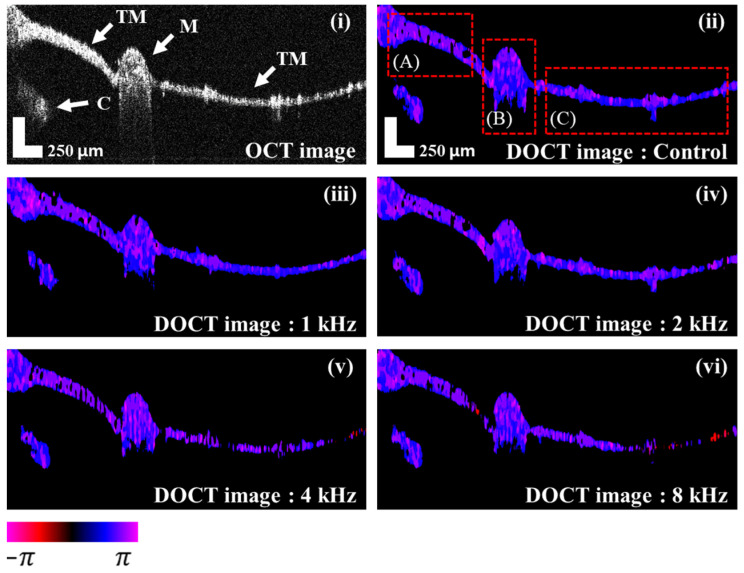
The OCT (optical coherence tomography) image and the DOCT images of the TM vibrating at each frequency. The rectangular boxes in (**A**–**C**) represent the ROI for phase change analysis. C: cochlea; M: malleus; TM: tympanic membrane.

Figure 3 shows the cross-sectional OCT image and cross-sectional DOCT images from the TM of a representative mouse sample in response to pure tone stimuli. The red square boxes are regions of interest (ROI) classified as thick TM, malleus, and thin TM, respectively. The DOCT images are shown for the measurements made in the absence of sound (control) and at 1, 2, 4, and 8 kHz. The malleus and the thick and thin sections of the TM were confirmed by the OCT image, and the end of the cochlea under the TM was also confirmed. From the Doppler OCT images, it was confirmed that more magenta and red colors were distributed in the TM as the frequency increased. In the malleus, vibration was more sensitive than TM at high frequencies. There are three reasons why the phase shift signal may disappear slightly at 4 and 8 kHz ROI (**C**). First, since the camera line period is set to 100 μs. Therefore, according to the Nyquist theorem, the phase shift can be reduced due to the effect of aliasing in the stimulated frequencies which exceed 5 kHz. The second reason could be due to signal attenuation by fringe washout [33]. As the sample oscillates rapidly, the number of charged electrons in the image sensor may be too low to attenuate the signal. Finally, when the phase shift is shifted by 2 π, the output color is black. In this case, we excluded this method to avoid more complexity and difficulties during phase unwrapping in the detected interference signals.

**Figure 4:**
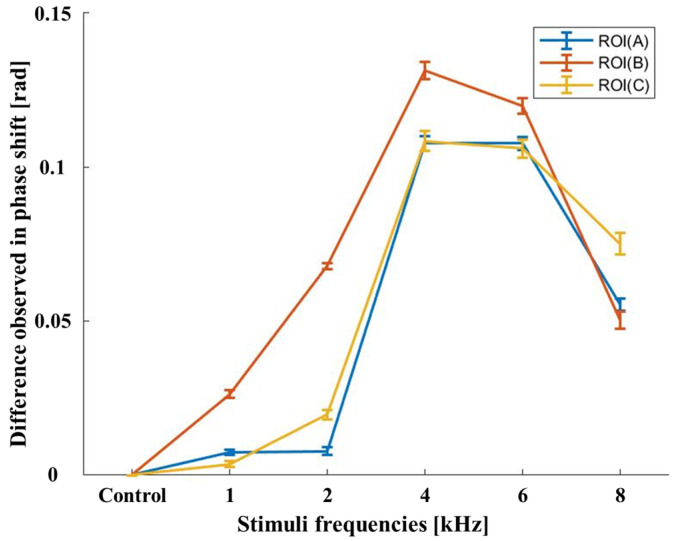
The average phase variation calculated in each ROI region according to the frequency.

Figure 4 shows the average of the phase shifts within each ROI (**A**–**C**). The DOCT image of the control sample in Figure 3 (ii) shows changes in phase according to sample structure. The error bar denotes the maximum and minimum measured values that were obtained for the three different ROI (shown as red, yellow, and blue plots) within each tympanic membrane for all 10 samples. Furthermore, we did not apply any external sound stimuli to the sample during this state. This change in phase for individual sections of samples observed in DOCT images was taken to be an initial reference value for each ROI in the image. When the external sound stimulus was applied for each selected frequency, we recorded the DOCT image containing the difference in phase value in each ROI in the TM. This change in phase value of ROI in TM in accordance with different frequencies was calculated by subtracting the phase value recorded in the control sample (initial reference values) and it was plotted as shown in Figure 4 for easier representation of the observed results. The phase shift also increased with increasing frequencies of up to 4 kHz. The Doppler phase shift of the 4 kHz stimulus was the greatest in all ROI. In particular, the signal was larger in the malleus in the ROI (**B**), which received sound vibrations from the TM and delivered it to the cochlea. At 6 and 8 kHz, the phase shift was decreased, and the decrease was largest in the malleus. At 8 kHz pure tone stimuli, the difference in Doppler phase shift was observed to decrease, and the total drop-off of ROI (**B**) is considerably larger when compared to ROI (**A**) and ROI (**C**). A thick part of the TM was more responsive at a low frequency of 1 kHz and was almost similar at frequencies of 4 kHz and 6 kHz. At high frequencies of 8 kHz, the thinner part of the TM was more responsive than the thicker part. The quantitative results in Figure 4 were averaged from dividing the sum of the Doppler shifts in the ROI in the DOCT images by the number of pixels with the Doppler signal. The reference value of the non-vibrating control value was used for quantitative comparison with the Doppler signals of the vibrating TM.

## 4. Conclusions

In general, conventional diagnostic tools such as audiometry, tympanometry, otoscopy, and LDV are used to diagnose the status of the TM. However, the devices used with these methods cannot structurally diagnose faults within the TM nor image vibrations. Recent OCT technology has been developed enough for applications in clinics other than for ophthalmology, and Doppler technology has developed along with it. 

DOCT is a technology used to image TM internal structures and vibrations; this technique is different from conventional methods in which the patient responds by observing the surface of the TM using a microscope or camera and outputting a small sound using headphones. This study has shown that DOCT can simultaneously diagnose middle ear structures and movements, including those of the TM and ossicular chain, in small animal models. Through these experiments we have shown that DOCT can be used in TM and in other biological samples, where vibratory phenomena of the samples needs to be measured in real-time.
